# Supplementary material for: Transcriptomic analyses reveal the potential regulators of the storage root skin color in sweet potato
Source: PeerJ. 2025 Dec 1;13:e20231. doi: 10.7717/peerj.20231 (PMC12677044; doi:10.7717/peerj.20231)
Supplement: Supplemental Information 7 [file peerj-13-20231-s007.docx]

>AtMYB3 NP_564176.2

MGRSPCCEKAHMNKGAWTKEEDQLLVDYIRKHGEGCWRSLPRAAGLQRCGKSCRLRWMNY

LRPDLKRGNFTEEEDELIIKLHSLLGNKWSLIAGRLPGRTDNEIKNYWNTHIKRKLLSRG

IDPNSHRLINESVVSPSSLQNDVVETIHLDFSGPVKPEPVREEIGMVNNCESSGTTSEKD

YGNEEDWVLNLELSVGPSYRYESTRKVSVVDSAESTRRWGSELFGAHESDAVCLCCRIGL

FRNESCRNCRVSDVRTH

>AAC83582.1 AtMYB4

MGRSPCCEKAHTNKGAWTKEEDERLVAYIKAHGEGCWRSLPKAAGLLRCGKSCRLRWINY

LRPDLKRGNFTEEEDELIIKLHSLLGNKWSLIAGRLPGRTDNEIKNYWNTHIRRKLINRG

IDPTSHRPIQESSASQDSKPIQLEPVTSNTINISFTSAPKVETFHESISFPGKSEKISML

TFKEEKDECPVQEKFPDLNLELRISLPDDVDRLQGHGKSTTPRCFKCSLGMINGMECRCG

RMRCDVVGGSSKGSDMSNGFDFLGLAKKETTSLLGFRSLEMK

>AtMYB6 NP_192684.1

MGRSPCCEKAHTNKGAWTKEEDQRLVDYIRNHGEGCWRSLPKSAGLLRCGKSCRLRWINY

LRPDLKRGNFTDDEDQIIIKLHSLLGNKWSLIAGRLPGRTDNEIKNYWNTHIKRKLLSHG

IDPQTHRQINESKTVSSQVVVPIQNDAVEYSFSNLAVKPKTENSSDNGASTSGTTTDEDL

RQNGECYYSDNSGHIKLNLDLTLGFGSWSGRIVGVGSSADSKPWCDPVMEARLSLL

>AtMYB24 NP_198851.1

MEKRESSGGSGSGDAEVRKGPWTMEEDLILINYIANHGEGVWNSLAKSAGLKRTGKSCRL

RWLNYLRPDVRRGNITPEEQLTIMELHAKWGNRWSKIAKHLPGRTDNEIKNFWRTKIQKY

IIKSGETTTVGSQSSEFINHHATTSHVMNDTQETMDMYSPTTSYQHASNINQQLNYGNYV

PESGSIMMPLSVDQSEQNYWSVDDLWPMNIYNGN

>AtMYB75 AAG42001.1

MEGSSKGLRKGAWTTEEDSLLRQCINKYGEGKWHQVPVRAGLNRCRKSCRLRWLNYLKPS

IKRGKLSSDEVDLLLRLHRLLGNRWSLIAGRLPGRTANDVKNYWNTHLSKKHEPCCKIKM

KKRDITPIPTTPALKNNVYKPRPRSFTVNNDCNHLNAPPKVDVNPPCLGLNINNVCDNSI

IYNKDKKKDQLVNNLIDGDNMWLEKFLEESQEVDILVPEATTTEKGDTLAFDVDQLWSLF

DGETVKFD

>AtMYB90 NP_176813.1

MEGSSKGLRKGAWTAEEDSLLRLCIDKYGEGKWHQVPLRAGLNRCRKSCRLRWLNYLKPS

IKRGRLSNDEVDLLLRLHKLLGNRWSLIAGRLPGRTANDVKNYWNTHLSKKHESSCCKSK

MKKKNIISPPTTPVQKIGVFKPRPRSFSVNNGCSHLNGLPEVDLIPSCLGLKKNNVCENS

ITCNKDDEKDDFVNNLMNGDNMWLENLLGENQEADAIVPEATTAEHGATLAFDVEQLWSL

FDGETVELD

>IbMYB1 AGE92356.1

MVISSVWSGSSSRVRKGSWSEEEDQLLRECIQKYGEGKWHLIPLRAGLNRCRKSCRLRWL

NYLRPDIKRGEFSPDEIDLILRLHRLLGNRWSLIAGRIPGRTANDVKNLWNTHLQKKVSA

MASSRQDNYWKGKAPEITENTVVRPRPRRFLKASSSPTTLLTGNATMVAYDGQLQEHMTT

QPETTSDLLMENVQLKNLTTTLPSALETTPHDNVKWWEDVLSDKELNEEGQICWSEFPTD

IDLLSELLS

>AtMYB113 NP_176811.1

MGESPKGLRKGTWTTEEDILLRQCIDKYGEGKWHRVPLRTGLNRCRKSCRLRWLNYLKPS

IKRGKLCSDEVDLVLRLHKLLGNRWSLIAGRLPGRTANDVKNYWNTHLSKKHDERCCKTK

MINKNITSHPTSSAQKIDVLKPRPRSFSDKNSCNDVNILPKVDVVPLHLGLNNNYVCESS

ITCNKDEQKDKLININLLDGDNMWWESLLEADVLGPEATETAKGVTLPLDFEQIWARFDE

ETLELN

>FaMYB1 AAK84064.1

MRKPCCEKTETTKGAWSIQEDQKLIDYIQKHGEGCWNSLPKAAGLRRCGKSCRLRWINYL

RPDLKRGSFGEDEEDLIIRLHKLLGNRWSLIAGRLPGRTDNEVKNYWNSHLKKKILKTGT

TLRPNKPHENNHAPNNKLVKLFNKMDDEVVDEVSSADSAAGCLVPELNLDLTLSIKTSTG

MADPQVA

>FcMYB1 ADK56163.1

MRKPCCEKTETTKGAWSIQEDQKLIDYIQKHGEGCWNSLPKAAGLRRCGKSCRLRWINYL

RPDLKRGSFGEDEEDLIIRLHKLLGNRWSLIAGRLPGRTDNEVKNYWNSHLKKKILKTGT

TLRPNKPHENNHAPNNKLVKLFNKMDDEVVDEVSSADSAAGCLVPELNLDLTLSIKTSTG

MADPQVA

>FaMYB1-1 MG456857

MRKPCCEKTETTKGAWSIQEDQKLIDYIQKHGEGCWNSLPKAAGLRRCGKSCRLRWINYL

RPDLKRGSFGEDEEDLIIRLHKLLGNRWSLIAGRLPGRTDNEVKNYWNSHLKKKILKTGT

TLRPNKPHENNHAPNNKLVKLFNKMDDEVVDEVSSADSAAGCLVPELNLDLTLSIKTSTG

MADPQVA

>FaMYB1-2 MG456858

MRKPCCEKTETTKGAWSIQEDQKLIDYIQKHGEGCWNSLPKAAGLRRCGKSCRLRWINYL

RPDLKRGSFGEDEEDLIIRLHKLLGNRWSLIAGRLPGRTDNEVKNYWNSHLKKKILKTGT

TLRPNKPHENNHAPNNKLVKLFNKMDDEVVDEVSSADSAAGCLVPELNLDLTLSIKTSTG

MADPQVA

>FaMYB9 AFL02460.1

MGRSPCCSKEGLNRGAWTALEDKVLTSYIKAHGEGKWRNLPKRAGLKRCGKSCRLRWLNY

LRPDIKRGNISGDEEELIIRLHNLLGNRWSLIAGRLPGRTDNEIKNYWNTTLSKKAKPES

HSGSSKETSPGPTRFRPRKASAAATTQPQVIRTKATRLTRMPVPSLPLLIDDCSTSTTAL

ELQVPQTQLVSSLPEDAVNTQVHFQGTDAMNFGCNGFQATAGDDEDAKGDYDIPLDDGML

NDWTGNGNCDLENYGASLDLDSLAFLLDSDD

>FaMYB10 ABX79947.1

MEGFGVRKGAWTKEEDELLKQFIEIHGEGKWHHVPLKSGLNRCRKSCRLRWLNYLKPNIK

RGEFAEDEVDLIIRLHKLLGNRWSLIAGRLPGRTANDVKNYWNTYQRKKDQKTASYAKKL

KVKPRENTIAYTIVRPRPRTFIKRFNFTERYANIEHNHSEVSYTSSLPTEPPQTLQLENV

TDWWKDFSEDSTESIDRTMCSGLGLEDHDFFTNFWVEDMLLSASNDLVNISYV

>FvMYB10 ABX79948.1

MEGYFGVRKGAWTKEEDELLKQFIEIHGEGKWHHVPLKSGLNRCRKSCRLRWLNYLKPNI

KRGEFAEDEVDLIIRLHKLLGNRWSLIAGRLPGRTANDVKNYWNTYQRKKDQKTASYAKQ

LKVKSQENTKATTIVRPRPRTFIKRFNFTERYENIEHNHSEMSYTSSLPTAPPQTLQLEN

VTDWWKDFAEDSTESIDRTMCSGLIGLEDHDFFTNFWVEDTVQSASNDLVNISYV

>FaMYB10-1 MG456859

MEGFGVRKGAWTKEEDELLKQFIEIHGEGKWHHVPLKSGLNRCRKSCRLRWVNYLKPNIK

RGEFAEDEVDLIIRLHKLLGNRWSLIAGRLPGRTANDVKNYWNTYQRKKDQKTASYAKKL

KVKPRENTIAYTIVRPRPRTFIKRFNFTERDANIEHNHSEVSYTSSLPTEPPQTLQLENV

TDWWKDFSEDSTESIDRTMCSGLGLEDHDFFTNFWVEDMVLSASNHLVNISYV

>FaMYB10-2 MG456860

MEGYFGVRNGAWTKEEDELLKQFIEIHGEGKWHHVPLKSGLNRCRKSCRLRWLNYLKPNI

KRGEFAEDEVDLIIRLHKLLGNRWSLIAGRLPGRTANDVKNYWNTYQRKKDQKTASYAKK

LKVKPRENTIAYTIVRPRPRTFIKRFNFTERYANIEHNHSEVSYTSSLPTEPPQTLQLEN

VTDWWKDFSEDSTESIDRTMCSGLGLEDHDFFTNFWVEDMVLSASDDLVNISYV

>FaMYB11 AFL02461.1

MGRSPCCAKEGLNRGAWTAMEDRTLTEYITTHGEGKWRNLPKRAGLKRCGKSCRLRWLNY

LRPDIKRGNITRDEEELIIRLHKLLGNRWSLIAGRLPGRTDNEIKNYWNTNIRKKVQDHS

STNSEANITHHKPPNHQTQKKNTNVVRTKASRCTKVFMPHQQSQMDKKGTCNNPTADQQG

AAPFLNHDYYDPINYNDDPALRMMGITDTHHQESDDLSPFLNLEIDNENSNSCGFMVDFK

MDESFLSEFLNVDFSELYSSSTSTANGGDGVKAVINNSCGDNDHHELHSPDFGSSMAPII

DSEVDWLS

>FvMYB305-like XP_011468270.1

MDKKPCNSSSQDAEVRKGPWTMEEDLILINYIANHGEGVWNSLAKSAGLKRTGKSCRLRW

LNYLRPDVRRGNITPEEQLLIMELHAKWGNRWSKIAKHLPGRTDNEIKNYWRTRIQKHIK

QGDQSNSQGQSSDGTADQASTSKVGSSSTVGLDAMETTYPLCAPTASYPATLVQPPAHPN

VLPPGDSNQDNYWSMEDLWSMQLLNGE

>MdMYB1 ABK58136.1

MEGYNENLSVRKGAWTREEDNLLRQCVEIHGEGKWNQVSYKAGLNRCRKSCRQRWLNYLK

PNIKRGDFKEDEVDLIIRLHRLLGNRWSLIARRLPGRTANAVKNYWNTRLRIDSRMKTVK

NKSQEMRKTNVIRPQPQKFNRSSYYLSSKEPILDHIQSAEDLSTPPQTSSSTKNGNDWWE

TLLEGEDTFERAAYPSIELEEELFTSFWFDDRLSPRSCANFPEGQSRSEFSFSTDLWNHS

KEE

>MdMYB6-like XP_008374825.1

MRKPCYEKYEINKGAWSKQEDQKLIDYIQKHGEGCWNSLPHAAGLSRCGKSCRLRWINYL

RPDLKRESIREDEEDLIIRLHALLGNRWSLIAGRLPGRTDNEVKNYWNTHIRKKLLKMGS

TLDPKKPHHHNDPHLRKGTTATVPLLQPDTSSPISFALSSSDSMSTGAEIHSNKSGLPDL

NLDLSL

>MdMYB9 NP_001280749.1

MGRSPCCSKEGLNRGAWTALEDKILSSYIKAHGEGKWRSLPKRAGLKRCGKSCRLRWLNY

LRPDIKRGNISGDEEELIVRLHNLLGNRWSLIAGRLPGRTDNEIKNYWNTTLGKKSKVDS

FSGSSKETSLNPCKSIAKKKDVESKTSTAAAQPLVIRTKATRLTKILVPQNIPSDENYTA

AAANPLELQTQSAEKGGSTEEFPRTNAGDCSNILKNFGCDDDDIDAKGDQYCNEFQLLNS

IPLDEAMINDGCWTGGNGCDLEDYGASLDLDSLAFLLDSEEWPSQENVVV

>MdMYB10 ACQ45201.1

MEGYNENLSVRKGAWTREEDNLLRQCVEIHGEGKWNQVSYKAGLNRCRKSCRQRWLNYLK

PNIKRGDFKEDEVDLIIRLHRLLGNRWSLIARRLPGRTANAVKNYWNTRLRIDSRMKTVK

NKSQEMRKTNVIRPQPQKFNRSSYYLSSKEPILDHIQSAEDLSTPPQTSSSTKNGNDWWE

TLLEGEDTFERAAYPSIELEEELFTSFWFDDRLSPRSCANFPEGQSRSEFSFSTDLWNHS

KEE

>MdMYB11 NP_001280958.1

MGRSPCCSKDEGLNRGAWTAMEDKVLTEYIGNHGEGKWRNLPKRAGLKRCGKSCRLRWLN

YLRPDIKRGNITRDEEELIIRLHKLLGNRWSLIAGRLPGRTDNEIKNYWNTTIGKRIQVE

GRSCSDGNRRPTQEKPKPTLSPKPSTNISCTKVVRTKASRCTKVVLPHESQKFGYSTEQV

VNAAPTLDQAVNNPMVGIDDPLLPMSFLDDENNNSCEFLVDFKMDENFLSDFLNVDFSVL

YNNEGAGKAAAAATTEDTSNKLHGPDLRSSKAPIIESELDCWLVDN

>MdMYB16 ADL36756.1

MGRSPCCEKAHTNKGAWTKEEDDRLIAYIRAHGEGCWRSLPKAAGLLRCGKSCRLRWINY

LRPDLKRGNFTEEEDELIIKLHSLLGNKWSLIAGRLPGRTDNEIKNYWNTHIRRKLLTRG

IDPTTHRPLNETPQESATTISFAAASANIKEEDKKISITNGLVCKDSKNPVQERCPDLNL

DLQISPPCQPQQPSDGLKSGGRGLCFSCSLGLQDAKNCSCGRDAIGGATSGTTNIGYDFL

GLKNGVLDYRSLEMK

>MdMYB17 ADL36757.1

MRKPCCEKKKTNKGAWSKQEDEKLTEFVEKNGEGSWRSLPLAAGLLRCGKSCRLRWVNYL

RPNLKRGNFGEDEEDLIIKLHALLGNRWSLIAGRLPGRTDNEVKNYWNTHLRRKLIQMGV

DPNNHRIGHTQNIGLSKSSFGSRKANHPCKAANSQGDNDSDDHQKPFTDSASGPESNTSC

SGLPDLNLDLTIGLPS

>MdMYB21 NP_001280981.1

MAAPTTPNEENEFRRGPWTLEEDNLLIHYIVNHGEGHWNSVAKLAGLKRTGKSCRLRWLN

YLKPDIKRGNLTPQEQLMILELHSKWGNRWSKIAQHLPGRTGNEIKNYWRTRVQKQARQL

NIESNSEQFLDAVRGFWVPTLLQKMEQSSSSCSSTLSTSQNSASPCLSPNHAAPSVPLST

SPPSNATNVLDNYHISGNSNLATVPSNILSADSFVSHVPQMAEPSTSFPPAYYRLGYSSL

SPDGSHYVDSSSYDVEGLSLDPVSPMGNLGNSQFDCQMGGNDWMLDNVTDSLWNMDGP

>MdMYB111 ADL36754.1

MRKPCCEKEGTNKGAWSKQEDQKLIDYIKTHGEGCWRSLPKAAGLHRCGKSCRLRWINYL

RPDIKRGNFEQDEEELIIKLHALLGNRWSLIAGRLPGRTDNEVKNYWNSHIRKKLIKMGI

DPNNHRLNQIIPRPNPQNDSVSPAATSSGSMSNINACTKTPLKSSDDQIDHRASEAASVL

EDETSGPSSRDLNLDLTIAFPEPSLQVEEGMPKLIKGSNTTAREIETNLQHLPTLVLFR

>MdMYB305-like XP_008341440.1

MDKKPCNSSSQDVEVRKGPWTMEEDLILINYIANHGEGVWNSLAKSAGLKRTGKSCRLRW

LNYLRPDVRRGNITPEEQLLIMELHAKWGNRWSKIAKHLPGRTDNEIKNYWRTRIQKHIK

QAENITPGQSSEVNDQASTSQVSISNTVDTMDISHSAPTYQANMDAYPPPLPADQSNESY

WSMEDLWSLQLLNGE

>MsMYB10 ABX71485.1

MEGYNENLSVRKGAWTREEDNLLRQCVEIHGEGKWNQVSYKAGLNRCRKSCRQRWLNYLK

PNIKRGDFKEDEVDLIIRLHRLLGNRWSLIARRLPGRTANAVKNYWNTRLRIDSRMKTVK

NKSQEMRKTNVIRPQPQKFNRSSYYLSSKEPILDHIQSAEDLSTPPQTSSSTKNGNDWWE

TLLEGEDTFERAAYPSIELEEELFTSFWFDDRLSPRSCANFPEGQSRSEFSFSTDLWNHS

KEE

>ParMYB10 ABX71490.1

MEGYNLGVRKGAWTREEDDLLRQCIEKQGEGKWHQVPYKAGLSRCRKSCRLRWLNYLKPN

IKRGDFMEDEVDLIIRLHKLLGNRWSLIARRLPGRTANDVKNYWNTRLRTDYCMKKMKDK

SQETIKTIIRPQPRSFTKSSNCLSFKEPILDHTQLEEKFSETSQTSTSTRIGSDWWETFL

DDKDATETATGSGLGLDEEQLASFWVDDDMPQSTRTCINFSKEGLSRGDFSFSVDLWNHS

KEE

>PavMYB10.1 ALM31951.1

MEGYNLGVRKGAWTKEEDDVLRQCIENHGEGKWYQVPYKAGLNRCRRSCRLRWLNYLKPN

IKIGGFAEDEVDLIIRLHKLLGNRWSLIAGRLPGRTANDVKNYWNTRLRTDSRLKKVKDK

PQETIKTIVIRPQPQSFIKSSNCLSSKEPILDHIQTVENCSTPSQTSPSTKNGNDWWETF

LGDEDAFERATCYGLALEEEGFTSFWVDDMPQSKRQCTNVQKD

>PavMYB11 ALH21142.1

MGRSPCCSKDEGLNRGAWTAMEDKILTEYITVHGEGKWRNLPKRAGLKRCGKSCRLRWLN

YLRPDIKRGNITRDEEELIIRLHKLLGNRWSLIAGRLPGRTDNEIKNYWNTTIGKKIQGH

PFSDGNRKPPKQTQENPKPTQPPKVDTNSCTKVVRTKASRCTKVFIPQEAQNPDDHLGDD

NDHVSNNAPLVSVDQVNDQVAGIEEPLSPIFLLDDENSSCEFMVDFKVDENFLSDFLNVD

FSELYNNGNDEGGEVASATACDKVPDFRSSSMVPVIDYELDWLIDNTAH

>PavMYB111 ALH21138.1

MRKPCCEKEGTNKGAWSKQEDQKLIDYIKSHGEGCWRSLPKAAGLHRCGKSCRLRWINYL

RPDIKRGNFEQDEEDLIIKLHALLGNRWSLIAGRLPGRTDNEVKNYWNSHIRKKLIKMGI

DPNNHRLNQIIPRPNPQNDCVSAAATSSGSMSNISACTKAPIKSSREIDQRASQATSVLE

DETSGSSSRDLNLDLTIAFPNPPLQVEEEMQKNIKGSFTMAREIETTLQHSPTLALFR

>PavMYBR ADY15315.1

MGRSPCCEKAHTNKGAWTKEEDDRLIAYIRAHGEGCWRSLPKAAGLLRCGKSCRLRWINY

LRPDLKRGNFTEEEDELIIKLHSLLGNKWSLIAGRLPGRTDNEIKNYWNTHIRRKLLTRG

IDPTTHRALNEAAQDSATTTISFAASANIKEEDQKSSIINGLLGKDSKKPVQERCPDLNL

ELQISPPCQPQQPSEPLKSGGRGVCFSCSLGLQDAKNCSCGIDTIGSSTTSGTTNVGYDF

LGLKSGVLDYRSLEMK

>PcfMYB10 ABX71495.1

MEGYNLGVRKGAWTRKEDDLLRQCIEKHGEGKWHQVPYKAGLSRCRRSCRLRWLNYLKPN

IKRGDFMEDEVDLIIRLHKLLGNRWSLIARRLPGRTANDVKNYWNTRLRKDYCMKKMKDK

SQETIKTIIRPQPRSFTKSSNCLSFKEPILDHTQLEENFSTPSQTSTSTRIGSDWWETFL

DDKDATERDTGSGLGLDEELLASFWVDDDMPQSTRTCVNFSEEGLSRGDFSFSVDLWNHS

KEE

>PdmMYB10 ABX71492.1

MEGYNLGVRKGAWTREEDDLLRKCIEKQGEGKWHQVPYKAGLSRCRKSCRLRWLNYLKPN

IKRGDFMEDEVDLIIRLHKLLGNRWSLIARRLPGRTANDVKNYWNTRMKKMKDKSQETIK

TIIRPQPRRFTKSSNCLSFKEPILDHTQLEENFSTTSQTSTSTRIGSDWWETFLDDKDAT

ETATGSGLGLDEELLASFWVDDDMPQSTRTCVNFSEEGLSRGDFSFSVDLWNHSKEE

>PpyMYB10 ABX71488.1

MEGYNVNLSVRKGAWTREEDNLLRQCIEIHGEGKWNQVSYKAGLNRCRKSCRQRWLNYLK

PNIKRGDFKEDEVDLILRLHRLLGNRWSLIARRLPGRTANDVKNYWNTRLGIDSRMKTLK

NKSQETRKTNVIRPQPQKFIKSSYYLSSKEPILEHIQSAEDLSTPSQTSSSTKNGNDWWE

TLFEGEDTFERAACPSIELEEELFTTFWFDDRLSARSCANFPEEGQSRSEFSFSMDLWNH

SKEE

>VvMYBA1 BAE96751.1

MESLGVRKGAWIQEEDVLLRKCIEKYGEGKWHLVPLRAGLNRCRKSCRLRWLNYLKPDIK

RGEFALDEVDLMIRLHNLLGNRWSLIAGRLPGRTANDVKNYWHSHHFKKEVQFQEEGRDK

PQTHSKTKAIKPHPHKFSKALPRFELKTTAVDTFDTQVSTSSKPSSTSPQRNDDIIWWES

LLAEHAPMDQETDFSASGEMLIASLRTEETATQKKGPMDGMIEQIQGGEGDFPFDVGFWD

TPNTQVNHLI

>VvMYB6 XP_002273328.1

MRKPCCDKKDTTKGAWSKQEDQRLIDYIKTHGEGCWRSLPKAAGLHRCGKSCRLRWINYL

RPDLKRGNFGQDEEDLIIKLHALLGNRWSLIAGRLPGRTDNEVKNYWNSHIRKKLINMGI

DPNNHRVNQSFAAPLNRCASAATMSSGSMTGACDNKSLKPSADNDPLSDSASGLEDEISA

SLDLNLDLTIAIPASSLTDVIDKKRQDTKSIFSREVEGDANPTLILFR

>VvMYB24 NP_001268062.1

MDKKPCNSQDAEVRKGPWTLGEDLILINYIANHGEGVWNSLAKSAGLKRTGKSCRLRWLN

YLRPDVRRGNITDEEQQLIMELHAKWGNRWSKIAKHLPGRTDNEIKNFWRTRIQKHIKNA

ETFTAQSSDQTHDQATTSQVMGAAHVADSYSPPSYPANLEAFPGPSSAESNDNFWTMEDI

WSMQLLNGD

>VvMYB308 XP_010648383.1

MGRSPCCSKEGLNRGAWTALEDKILTAYIKAHGEGKWRNLPKRAGLKRCGKSCRLRWLNY

LRPDIKRGNISHDEEELIIRLHKLLGNRWSLIAGRLPGRTDNEIKNYWNTTLGKKIGAQP

TNQSRLKSKPPIDHKPTAIEPEAAPAQPQVIRTKATRCTKVLVPTDPPPRLSEPRPIDST

TPQNPLQAQPQQTHSVAPWGPTDFTPDYGTITNSNLFNEDYSYSNLLENVTPFKFEDWAS

NDCLENNAALDLDSLAFLLNSEEWP

>Ibat.Brg_v3.09FG001230.1

MGRSPCCEKAHTNKGAWTKEEDERLIAYIRAHGEGCWRSLPKAAGLLRCGKSCRLRWINY

LRPDLKRGNFTEEEDELIIKLHSLLGNKWSLIAGRLPGRTDNEIKNYWNTHIRRKLLSRG

IDPTTHRPVDDPKEKVTTISFGPAKAAQEDDVNVVDEKKMMMIRQDSLPTTTTTTTTVKQ

ESTSPVRDDDERCPDLNLELRISPPYHPNQQPPLTLTPGSLVNVNNTAVCFACSLGIQNS

KDCTCSTNANAITSIAGYDFLGLRKNTLLDYRNLETMMN*

>Ibat.Brg_v3.07AG005840.1

MRKASCDHSHHHHHEINKGAWSKQEDQKLLDYIRKHGEGGWRDLPKAAGLLRCSKSCRLR

WMNHLKQTAKRGNFGDDEEDLIIKLHALLGDRWSLIAGRLPGRTEEEVKNYWNSHIKKKL

LDMGIDPNNHRLSCTYSRPHNIAAQTSAGKSRVTLPEKQRVENDGEVADAGSSNVR*

>Ibat.Brg_v3.03EG020400.1

MVRAPCCEKMGMKKGPWTPEEDQILTSFIQRYGHENWRALPRQAGLLRCGKSCRLRWINY

LRPDIKRGNFSKDEEETIIQLHQTLGNRWSAIASRLPGRTDNEIKNFYNTHLKKRLQHHG

SPYYSPNNVVGNITPIQIGDSSIHLRFPAPMTVNCNPVHRQNSTYSISSPMTTKMEEEES

MQESYQNLGATSNDDSGIVYLPSSSSVLPMELGGCETSSSISNDAVFWYNLLINAGNTS*

>Ibat.Brg_v3.04FG035800.1

MVQEEFRKGPWTEQEDVQLVFYVNLFGDRRWDFLAKVSGLEGLKRTGKSCRLRWVNYLHP

GLKRCKMTPQEERLILELHSKWGNKWSRIARKLPGRTDNEIKNYWRTHMRKKAQENKKKK

GGACISASSSSLTNCCSYSSSANSPAAVESEVDEANERDFYDTGGIDEETTQVPPVQENG

GGAKAYTMDEIWKDIEQLGDVYCGNNQQRSVTTSSPMWNYWAETLWMTTADYNHGGGESK

TVSFPPLPLPLPPPPPTNNDQFYSSFDNQGSIFLTG*

>Ibat.Brg_v3.08CG009710.1

MGRQPCCDKLGVKKGPWTAEEDKKLITFILSNGQCCWRALPKLAGLRRCGKSCRLRWTNY

LRPDLKRGLLSQDEEQLVIDLHARLGNRWSKIASKLPGRTDNEIKNHWNTHIKKKLIKMG

IDPVTHEPLIKDTTNPTSDKGNGQQQVQVVPEGTPPTAPNLTSEDLSSPCSTSENSSITS

TNDDSQLVLDTMSDNDPLLSSLLENNAPPVDLTWSLSDDQMIFDNLTIPKLDENFAWLMD

GQDFGIHDFGYECSNNLDVNILSTVNTQNNVQ*

>Ibat.Brg_v3.12FG016950.2

MGRAPCCSKEGLKKGPWSTKEDLLLTNYIQQHGEGQWRSLPKKAGLLRCGKSCRLRWMNY

LRPGIKRGNFSPEEEDLIVRLHSLLGNRWSLIAGRLPGRTDNEIKNYWNTHLLKKLKSSG

IEPRPPRKIVTSKKKATIPKIVASKKPANNNSRNKKLQRKEISDDNQRCYKVYAPKAIRL

SSRNNSVEDVAGSVSSSSGEVENKGIIDGSSSFIPWNLYELRDDFCAEVLTAAGDDLSPQ

CALPVGSDDCLLDKVYDEYLQLLSENCFLEDDPFGANL*

>Ibat.Brg_v3.13CG020320.1

MDHVKGGGAYKSVAQQQAEDDADLRRGPWTVEEDFTLINYIAHHGEGRWNSLARCAGLKR

TGKSCRLRWLNYLRPDVRRGNITLEEQLLILELHSRWGNRWSKIAQHLPGRTDNEIKNYW

RTRVQKHAKQLKCDVNSKQFKDTMRYLWMPRLMERIQAAAANAAVSSSTTTSANSDAAAY

IPLDISATSDPPAAGMQQHPTNAAPTIPDYNAAAVNFPSDNSSTAASSDSFSDLTDCCGY

NFHVTQGANHDYYQPNNNNHFGYGESLTSPTGFFNPNLDFQMMDNNNNNNNNNNQQWMDG

ADVVSDNLWNIEDMWFFQQQFNNNNNNSHP*

>Ibat.Brg_v3.12AG004780.1

MANSSCAWSGVRKGAWSEEEDNLLRKCIQKYGEGKWHLIPFRAGLNRCRKSCRLRWLNYL

RPDIKRGDFKLDEVDLILRLHKLLGNRWSLIAGRIPGRTANDVKNFWNTHIQKKVFAMAA

ASSDNWKGKAPEMRENTVVRPRPRRLSYRTPLTGKATAVICDAQIQGHKIPTSELVMENL

QENNTITSELETTTSNDKVQWWEDFLFDNEGSTCVNQGQVGWANFTIDMDLSQLLS*

>Ibat.Brg_v3.01BG005510.2

MGRAPCCDKDGLKRGPWTAEEDQKLIDYINKNGYGNWRTLPTNAGLQRCGKSCRLRWMNY

LRPDIKRGRFSSEEEHVIIQLHSILGNKWSAIAARLPGRTDNEIKNYWNTHIRKKLLRMG

IDPVTHRRRVDLLDLSSILNNPSFLYNSRILGAQTLYNVINSENANNVVQDTQQLHAPPP

LIQDFPVYSPNMAAAQITQQPNVEFGIENYPANDFWLPESEMTQDYLLPPLQNYGYYEAA

VDPQSAMDLPAPAADESCRFGFRQVWSTPSSSQVNSGSSTTTEDEREISYGSNLLNFDVE

NIFGVNEFM*

>Ibat.Brg_v3.12FG027390.1

MQVVAGTMRRPSSPTLSGSSGGRGDENAGGVKKGPWTPEEDKKLVDYIRKHGHGSWRAVP

KLAGLNRCGKSCRLRWTNYLRPDIKRGKFEEEEEQLIIKLHSVLGNKWSAIAMRLPGRTD

NEIKNHWNTHLRKRLLQMGIDPVTHRPRTDINFIDALANLPQLLVAAANMGNNSNVANPL

WDSINALRLCSDAAQIANELQLLQNFMALQLQLRGSVNNTTNEAQNQIPELATQFGSCNQ

LLDHLALLNPQLQGGLCNLGSSYNFSRLPPNISCSGSVATSSTSQNSEIQIHHPGIISNE

TNQGQTINSNVSRINDDSNKLMTNAFTVSSSSPLNVPSGEDIPSNPIFPTLIPASPFPEN

PSSSIDWETNKEKYTISANLKHDIPNHVPNATTTFEAWRDIKVDDDEATDSYWQDILYQT

FSP*

>Ibat.Brg_v3.12AG004740.1

MADSSSSEPPSGVKKGAWTEQEDNLLRKCIHKYGEGKWHLVPVRAGLNRCRKSCRLRWLN

YLRPDIKRGDFNLDEIDLIMRLHKLLGNRWSLIAGRIPGRTANDVKNLWNTRLQKKTIAN

NTPSSGQEKWKDKAPKATENTAVIRPRPRRFVMTSSSRTLPITGKTTIVTSEVVQLPPPP

PPAAETAESTSEPRLIENADPNNSIIDLAGETETSDDLGQWLDDFLFDMEFDGDGMACMQ

EGPIEWCDFHIDSDLLDLLN*

>Ibat.Brg_v3.01CG004930.1

MGRAPCCDKDGLKRGPWTAEEDQKLIDYINKNGYGNWRTLPTNAGLQRCGKSCRLRWMNY

LRPDIKRGRFSSEEEHVIIQLHSILGNKWSAIAARLPGRTDNEIKNYWNTHIRKKLLRMG

IDPVTHRRRVDLLDLSSILNNPSLLYNSRILGAQTLANPHLLRLAASQHHNNNVINSENA

NNVVQDTQQLHAPPLVQDFPVYSPNMAAAQITQQPNVEFGIENYPANDFWLPESEMTQDY

LLPPLQNYGYYEAAVDPQSAMDPPAAAADESCRFGFRQVWSTPSSNQVNSGSSTTTEDER

EISYGSNLLNFDVANIFGVNEFM*

>Ibat.Brg_v3.14FG021430.1

MGRSPCCEKMGLKKGPWTKEEDEILVDYISRHGHGNWRALPKHAGLLRCGKSCRLRWINY

LRPDIKRGNFSHEEEDAIIKLHQALGNRWSVIAARLPGRTDNEIKNIWHTRLKKRLNDYD

LVPSQPRLKSKSQPLKPFAMDLLITNGTLLTPSSPPHSSTTTSTDVHALSACSISSDCVV

SDTVLQSDPPEVDESFWSQVFSLENSSDVGDLPATVDGENRFDSTENETYETKSSVEFWH

RLFTKADDLPVLPEL*

>Ibat.Brg_v3.04AG030280.1

MVQEEFRKGPWTEQEDVQLVFYVNLFGDRRWDFLAKVSGLEGLKRTGKSCRLRWVNYLHP

GLKRCKMTPQEERLILELHSKWGNKWSRIARKLPGRTDNEIKNYWRTHMRKKAQENKKKK

GGACISASSSSLTNCCSYSSSANSPAAVESEVDEANERNFYDTGGDVEQLPPAHDNGGAG

AAKAYTMDEIWKDIEQLGDVYCGNNQPRSVTTSSPMWNYWAETLWMTTADYNPGAGGGES

KIVSSFPLPPPPTNDQFYSSFDNQGSIFLTG*

>Ibat.Brg_v3.13AG020090.1

MDHVKGGGAYKSVAQQQAEDDADLRRGPWTVEEDFTLINYIAHHGEGRWNSLARCAGLKR

TGKSCRLRWLNYLRPDVRRGNITLEEQLLILELHSRWGNRWSKIAQHLPGRTDNEIKNYW

RTRVQKHAKQLKCDVNSKQFKDTMRYLWMPRLMERIQAAAANAAVSSSTTTSANSDGAAY

IHNPLDISATSDPAGMQQHPNNAAPTIPDYNAAAAAAVNFPSDNSSTAASSDSFSDLTDC

CGYNFHVTQGANHDYYQPNNNNHLGYGESLTSPTGFFNPNLDFQMMDVNNNNNNNNNNNN

HQWMDGADVVSDNLWNIEDMWFSFQQQFNNNNNNSHP*

>Ibat.Brg_v3.07AG007770.1

MGRTPCCEKVGLKRGRWTAEEDRILTDYIHANGEGSWRSLPKNAGLLRCGKSCRLRWINY

LRSDLKRGKFSPEEEEIIIKSHAILGNRWSMIAAQLPGRTDNEIKNYWNSHLSRKFYSFR

RAGSEKTIENLKTDLAKAAEQTKRRRGKVSRSAMKKNKTTGYKHSSTINHPPELPHFPTL

CTEPVNNNAATSAVQIPSTPPILMEKEDDIDTSSFLCMAGSEYFSLDDIMPILVEEMDPG

TILSTSLGNNVKEFGVVAQSQHDDLAKNSNSSVHIYSEFIPSDHHHQFGGENSESTATSS

SFPVEHHCSLTQNIIDWDNWQYYWDDDSGNNLCNTQNLMPQQNDEDDVMLSSPWPWDDTF

YT*

>Ibat.Brg_v3.04FG026450.1

MGRSPCCEKAHTNKGAWTKEEDQLLINYIRLHGEGCWRSLPKAAGLLRCGKSCRLRWINY

LRPDLKRGNFTQQEDDLIIKLHSLLGNKWSLIAARLPGRTDNEIKNYWNTQIKRKLISRG

IDPQTHRPLDSSAGAGTGTTKPENISVDLSSSAPSQEETKCSSGTTSEESNHQSLKDKQR

NEQMGGLDLSIGLALESLTRRKQLFKMDSKML*

>Ibat.Brg_v3.08CG008750.1

MGRAPCCSKEGLRKGPWSAKEDLLLTNHIQEHGEGQWRSLPKKAGLLRCGKSCRLRWMNY

LRPGIKRGNFSQEEEDLIVRLHSLLGNRWSLIAGRLPGRTDNEIKNYWNTHLLKKLKSAG

IEPKPRQAKKKPAKPRPDSQNLNPNKKKKQKARNAEQPLRLADQTTAPEKTQNRTKVYAP

KPIRLSPPGISRINSLEDVAGSVSSSSGEVDNKAAVTADPPPFIPWHLYELGGGGDVDFC

DQILDGCDLSSPKCSGPTTDGLLEKVYDEYLHLLSENCFESLTDDYLCDYPFVDDNAAPT

TSSNNLDLN*

>Ibat.Brg_v3.08EG009880.1

MGRQPCCDKLGVKKGPWTAEEDKKLITFILSNGQCCWRALPKLAGLRRCGKSCRLRWTNY

LRPDLKRGLLSQDEEQLVIDLHARLGNRWSKIASKLPGRTDNEIKNHWNTHIKKKLIKMG

IDPVTHEPLIKDTTNPTSDKGNGQLQVQVVPEGTPPTAPILTSEDLSSSCSTSENSSITS

TNDDSQLVLDTMRDNDPLLSSLLEHNAPPVDLTWNLSDDQMIFDNLTIPKLDENFAWLMD

GQDFGILDFGFECSNNLDVNILSIVNTQNNVQ*

>Ibat.Brg_v3.03DG023570.1

MQRERERRMVRAPCCEKMGMKKGPWTPEEDQILTSFIQRYGHENWRALPRQAGLLRCGKS

CRLRWINYLRPDIKRGNFTKDEEETIIQLHQTLGNRWSAIASRLPGRTDNEIKNFYNTHL

KKRLHHHGSSPYSPNNVVGNITPIQIGDSSFHLRFPAPMTVNCNPVHGHGGRQNSAYSSS

TMTTKMEEEESMQESYQNLGSINDDSGIVYLPSSSSVLPMELGGCETSSSISNDAVFWYN

LLINAGNTS*

>Ibat.Brg_v3.S106200.1

MSFKAFESSSSGAFRFPPPPGPATLGLEEPEKRSGEQSHRLCARGHWRPHEDSRLRELVA

KHGPQNWNLIAEKIPGRSGKSCRLRWFNQLDPRINTKAFTEEEEERLLTAHRMYGNKWAI

IARLFPGRTDNAVKNHWHVIMARRHRHQTTGGARRRHPRNQNNNNNTMDSNNLVQIKNLN

DESAASTCTDLSLSTSSSSSSKLPPNHFHLTGFGPTPTHYHQILHTTPGSVKAAEASGRR

PESSPDSLITASDSVANNNNYGSEAEMCGQNQTAIYDNKFNNNNNKMLFFDFLGVGAN*

>Ibat.Brg_v3.13AG020210.1

MDHVKGGGAYKSVAQQQAEDDADLRRGPWTVEEDFTLINYIAHHGEGRWNSLARCAGLKR

TGKSCRLRWLNYLRPDVRRGNITLEEQLLILELHSRWGNRWSKIAQHLPGRTDNEIKNYW

RTRVQKHAKQLKCDVNSKQFKDTMRYLWMPRLMERIQAAAANAAVSSSTTTSANSDGAAA

YIHNPLDISATSDQAGMQQHPNNAAPTIPDYNNAAAAAVNFPSDNSSTAASSDSFSDLSD

CCGYNFHVTQGANHEYYQPNNNNHFGYGESLTSPTGFFNPNLDFQMMDNNNNNNNNNNQQ

WMDGADVVSDNLWNIEDMWFFQQQFNNNNNNSHP*

>Ibat.Brg_v3.07FG007500.1

MRKASCDHSHHHHEINKGAWSKQEDQKLLDYIRKHGEGGWRDLPKAAGLLRCSKSCRLRW

MNHLKQTAKRGNFGDDEEDLIIKLHALLGDRWSLIAGRLPGRTDEEVKNYWNSHIKKKLL

DMGIDPNNHRLSCTHNTAAQTSGGKSRVTSPEKQRVESDGEVSDAGSSNVR*

>Ibat.Brg_v3.03BG001270.1

MGRPPCCDKANVKRGPWTPEEDAKILAYVASHGIGNWTLVPQKAGLNRCGKSCRLRWTNY

LRPDLKHDNFTPEEEACILELHKTIGSRWSLIAKHLPGRTDNDVKNYWNTKLKKKLKNMG

IDPLTHKPFAQVFAEFGKLSGLPSPSNQNALLKNTIKNEAVFEPEPRSFPTNAQNSRFVS

PEMHEQLQIQNTPLVHNFPQEPIQPPHSSPDTSFPHFASSQYCSSSYEQPLSQFLTSSSS

TPWNEFILQDPCPMPDTELPRQDSKFPGTFSSDDPMTPSVQGEAGPICGFTNEDITEGDH

GEASSSMAEDSFVENILARDRQMQLEYPQLLDGYFD*

>Ibat.Brg_v3.11BG014930.1

MGRPPCCDKVGVKKGPWTPEEDIILVSYIQEHGPGNWRAVPTNTGLLRCSKSCRLRWTNY

LRPGIKRGNFTDQEEKMIVHLQALLGNRWAAIASYLPQRTDNDIKNYWNTHLKKKLKKVE

GSSEDGQDGNSSSSSHQSISKGQWEKRLQTDIHMAKQALCDALSIYKPATPEPFQPVHAL

NPPVQPSSGSTYASSTENIARLLQTWTKPVQSRSNSETTIQSSLNNNPYLGPGSSSSPSE

GTALSSTAFDQTVFGFNSNMDENNTWVFPVESKPQTVAPQNGNTSNNLGTQLPLTFLEKW

LLDDANNVPAPDDLMDMGMGIDLF*

>Ibat.Brg_v3.14CG019660.2

MGRSPCCEKMGLKRGPWTKEEDEILVDYISRHGHGNWRALPKHAGLLRCGKSCRLRWINY

LRPDIKRGNFSHEEEDAIIKLHQALGNRWSVIAARLPGRTDNEIKNIWHTRLKKRLNDYD

LVPSQPRLKSKSQPLKPFAMDLLITNGTLLTPSSPPHSSTTTSTDVHALSACSISSDCVV

SDAVLQSDPPEVDESFWSQVFSLENSSDAGDLPATVDGANRFDSTENETYETKSSVEFWH

RLFSKAEDLPVLPEL*
