# Supplementary figures and images for: Transcriptomic analyses reveal the potential regulators of the storage root skin color in sweet potato
Source: PeerJ. 2025 Dec 1;13:e20231. doi: 10.7717/peerj.20231 (PMC12677044; doi:10.7717/peerj.20231)

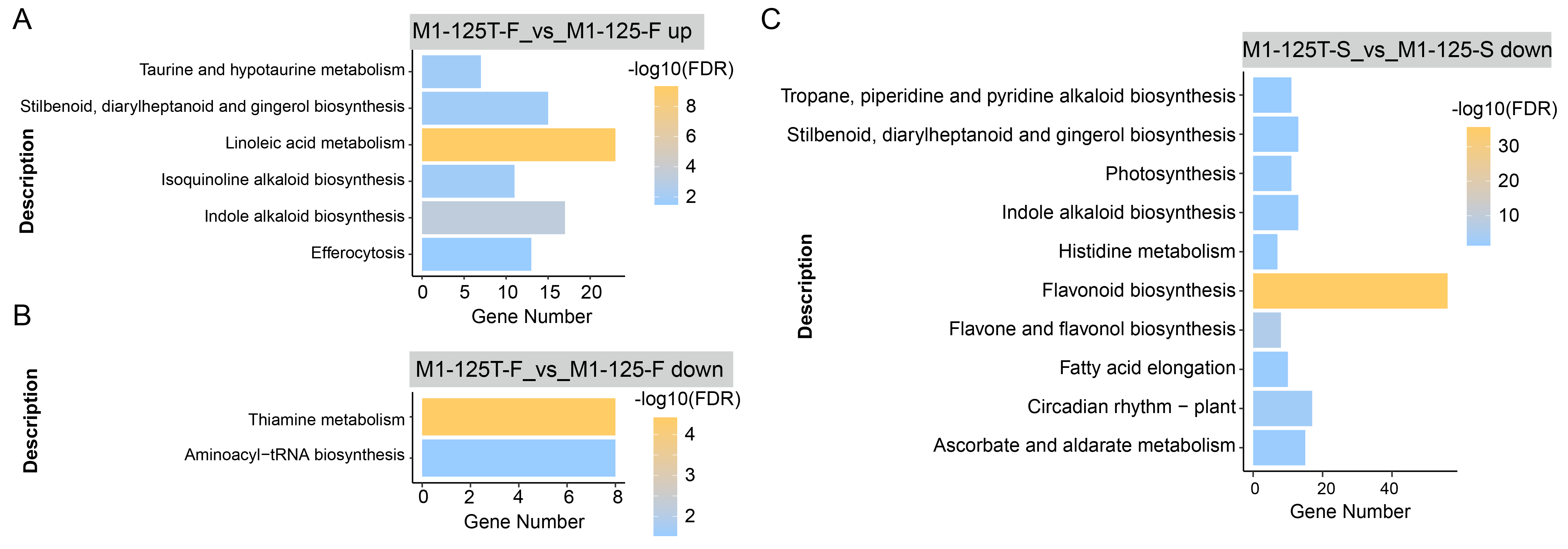

Supplement: Supplemental Information 9 — The enriched KEGG pathway of up-regulated genes (A) and down-regulated genes (B) in flesh, as well as down-regulated genes (C) in root skin, respectively. The color indicated the −log10(FDR) value, pathways with FDR < 0.05 were considered to be significantly enriched. [file peerj-13-20231-s009.png]

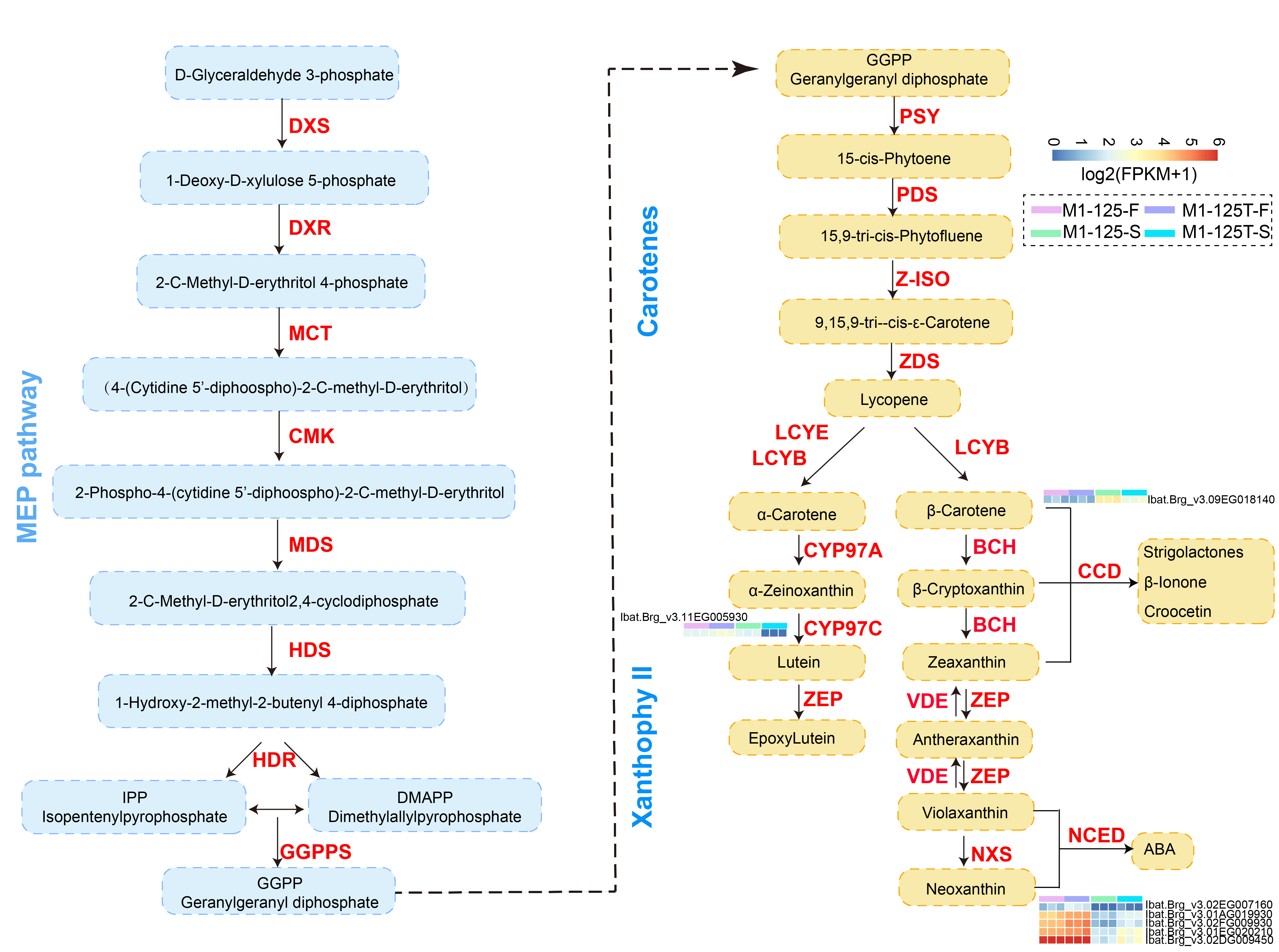

Supplement: Supplemental Information 10 [file peerj-13-20231-s010.png]

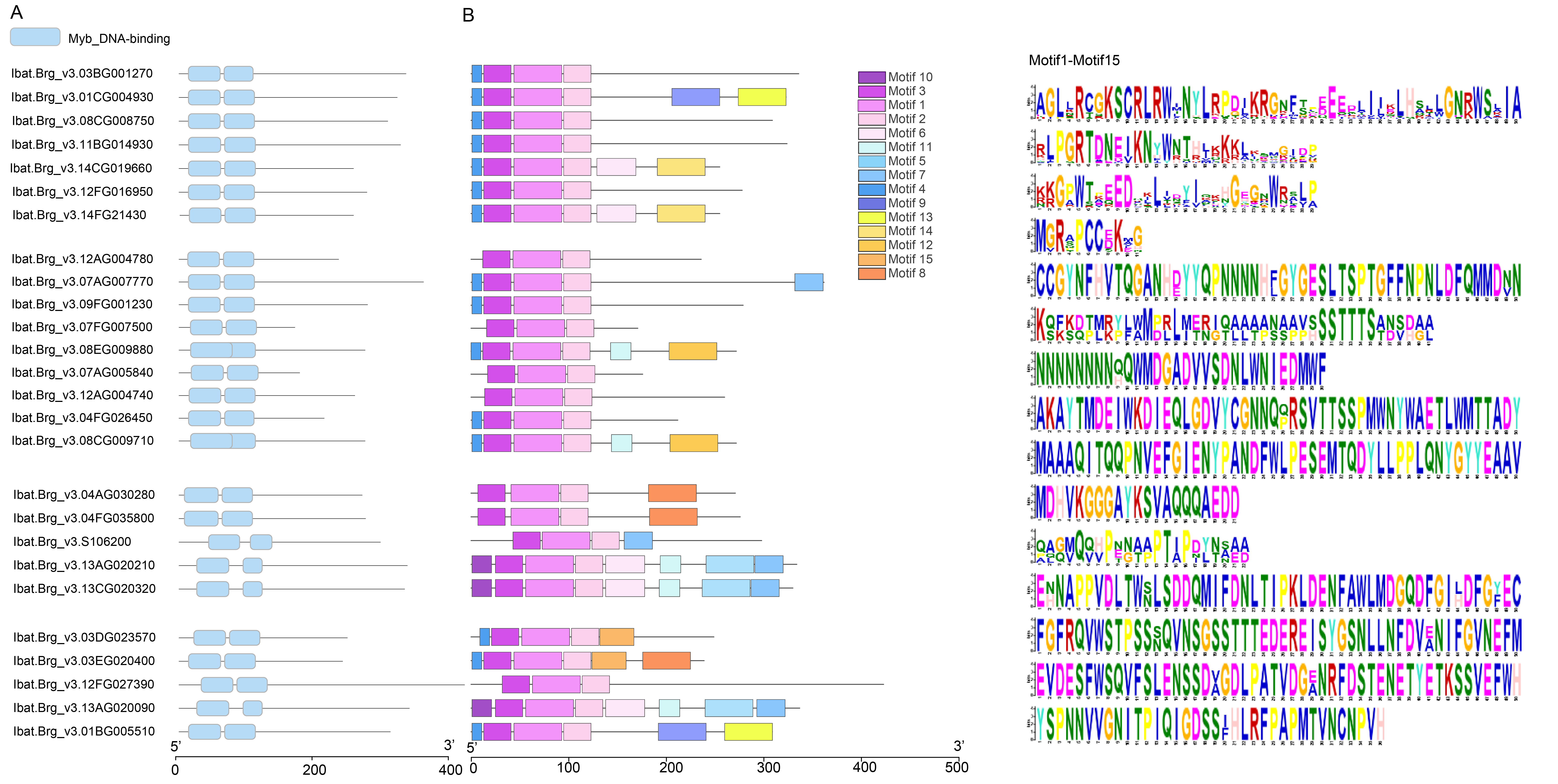

Supplement: Supplemental Information 11 [file peerj-13-20231-s011.png]
